# Rothia aeria Causing Prosthetic Valve Endocarditis in an Immunocompetent Patient

**DOI:** 10.7759/cureus.85286

**Published:** 2025-06-03

**Authors:** Kristy Leker, Dona T El-Khoury, Zayn Endo, Anderson Vu, Xolani Mdluli, Constantin A Dasanu

**Affiliations:** 1 Internal Medicine, Eisenhower Health, Rancho Mirage, USA; 2 Infectious Diseases, Eisenhower Health, Rancho Mirage, USA; 3 Internal Medicine/Infectious Diseases, Eisenhower Health, Rancho Mirage, USA; 4 Hematology Oncology, Eisenhower Health, Rancho Mirage, USA

**Keywords:** gram-positive endocarditis, immunocompetent, infective endocarditis, prosthetic valve endocarditis, rothia aeria

## Abstract

*Rothia aeria *is a part of the normal human oral flora and respiratory tract. This Gram-positive, pleomorphic rod has been linked to periodontal infections, typically occurring within immunocompromised hosts. To our knowledge, we present the first case of prosthetic valve endocarditis (PVE) secondary to *R. aeria *in an immunocompetent host. A 76-year-old man with a history of aortic valve replacement presented with generalized weakness and dyspnea on exertion eight weeks after deep dental cleaning for periodontal disease. He improved and was discharged from the hospital. Subsequently, blood cultures grew *R. aeria, *which was initially felt to be a contaminant. However, the patient returned a week later with similar symptoms, including night sweats and low appetite. Due to his recent normal outpatient transthoracic echocardiogram (TTE) and the concern for prosthetic valve endocarditis, we opted for a transesophageal echocardiogram (TEE). His TEE revealed a large aortic valve vegetation. Several repeat blood cultures grew again *R. aeria*. The patient responded to intravenous (IV) penicillin 24 million units q24 hours for six weeks and IV ceftriaxone 2 g IV q24 hours for two weeks. On a repeat TEE, there was a decrease in the size of the vegetation, and it resembled a calcified healed vegetation; the patient was able to return to his activities of daily living with significant symptomatic improvement. The rarity of severe disease due to *R. aeria* in immunocompetent hosts has likely been due to difficult diagnosis. With the advancement of matrix-assisted laser desorption ionization time-of-flight mass spectrometry (MALDI-TOF MS) (Bruker Microflex LT, Bruker Daltonics, Billerica, MA), *R. aeria *is now quickly and easily recognized. As our case suggests, *R. aeria *appears to be a true, non-contaminant bloodstream pathogen in both immunocompromised and immunocompetent hosts. Prompt diagnosis and aggressive treatment are imperative in order to ensure the best clinical outcomes.

## Introduction

*Rothia aeria* is a commensal microorganism native to the human oral flora and upper respiratory tract [1-9]. It is commonly considered a contaminant when collected in immunocompetent patients. Within immunocompromised patients, this Gram-positive, pleomorphic rod is often linked to periodontal infections; however, growing literature has seen *R. aeria* to cause more severe infections, including bacteremia and native valve endocarditis (NVE). On the other hand, prosthetic valve endocarditis (PVE) in immunocompetent hosts accounts for 20% of all cases of infective endocarditis and is associated with higher mortality compared to NVE [10]. Herein, we describe a rare case of an immunocompetent patient with PVE secondary to *R. aeria*, highlighting its potential as a bloodstream pathogen in immunocompetent hosts. We present therapies that may lead to treatment guidelines and illustrate the importance of further investigations.

## Case presentation

A 76-year-old man presented with a two-week history of generalized weakness and dyspnea on exertion. He was afebrile with night sweats and a low appetite. Past medical history was significant for coronary artery disease, calcific aortic stenosis, hypertension, hyperlipidemia, and periodontal disease. Past surgical history included coronary artery bypass surgery with simultaneous aortic valve replacement with a 25 mm Trifecta bioprosthetic valve in 2014.

Of note, he reported deep dental cleaning for periodontal disease eight weeks prior to presentation. Prior to his dental procedure, the patient took four 500 mg tablets of amoxicillin (2,000 mg in total) for prophylaxis against infective endocarditis, given his aortic valve replacement. He has no history of intravenous (IV) drug use, EtOH excess, or tobacco use.

On initial physical examination, he had a temperature of 37.3°C, a regular heart rate of 69 bpm, blood pressure of 149/76 mm Hg, respiratory rate of 15 breaths per minute, and O2 saturation of 97% on room air. He was alert and oriented, had pallor, but no splinter hemorrhages. On auscultation, a regular rate and rhythm were noted, with normal S1 and S2, and a 3/6 systolic murmur at the base. There was no lymphadenopathy, hepatomegaly, or splenomegaly. His blood counts were unremarkable. Two aerobic blood cultures (BACTEC FX, BD Diagnostics, Sparks, MD) were taken 15 minutes apart upon admission. During initial admission, the patient improved with intravenous hydration and was discharged the following day. The index blood cultures were flagged positive with a Gram-positive bacillus. *Rothia aeria* was later identified by matrix-assisted laser desorption ionization time-of-flight mass spectrometry (MALDI-TOF MS) (Bruker Microflex LT, Bruker Daltonics, Billerica, MA) following 48 hours of standard incubation.

Given that *R. aeria* was considered a commensal of the human oral flora, the index cultures were considered to reflect a contamination rather than an invasive infection. Following discharge, the patient continued to exhibit generalized weakness and decreased appetite with intermittent chills. One week later, he re-presented with a temperature of 36.9°C, a regular heart rate of 80 bpm, blood pressure of 109/67 mm Hg, respiratory rate of 19 breaths per minute, and O2 saturation of 93% on room air. Physical examination was unchanged. A complete blood count was remarkable for leukocytosis of 13.7 K/uL (normal range: 3.8 K/uL-10.8 K/uL), neutrophilia with an absolute neutrophil count of 12.0 K/uL (normal range: 1.6-7.8 K/uL), and lymphopenia with an absolute lymphocyte count of 0.7 K/uL (normal range: 1.2-3.7 K/uL). The patient had an elevated C-reactive protein of 6.7 mg/L (normal range: 0-1 mg/L) and a sedimentation rate of 105 mm/hour. Comprehensive metabolic panel revealed an elevated creatinine of 1.7 mg/dL (normal range: 0.7-1.3 mg/dL) and elevated liver enzymes with alkaline phosphatase of 506 IU/L (normal range: 34-104 IU/L), aspartate aminotransferase of 197 U/L (normal range: 13-39 U/L), and alanine aminotransferase of 375 IU/L (normal range: 7-52 IU/L).

There was no evidence of an immunocompromised state in this patient. Peripheral blood flow cytometry was unremarkable, except for slightly decreased CD4+ T-cells at 322/mm^3^ (normal range: 422-1,622/mm^3^), consistent with an acute infectious episode. Serum IgG, IgM, and IgA levels were normal. This laboratory work suggested no concern for underlying immunodeficiencies. He had a normal electrophoretic pattern with no monoclonal proteins noted on immunofixation electrophoresis, suggesting no concern for underlying malignancy.

**Figure 1:**
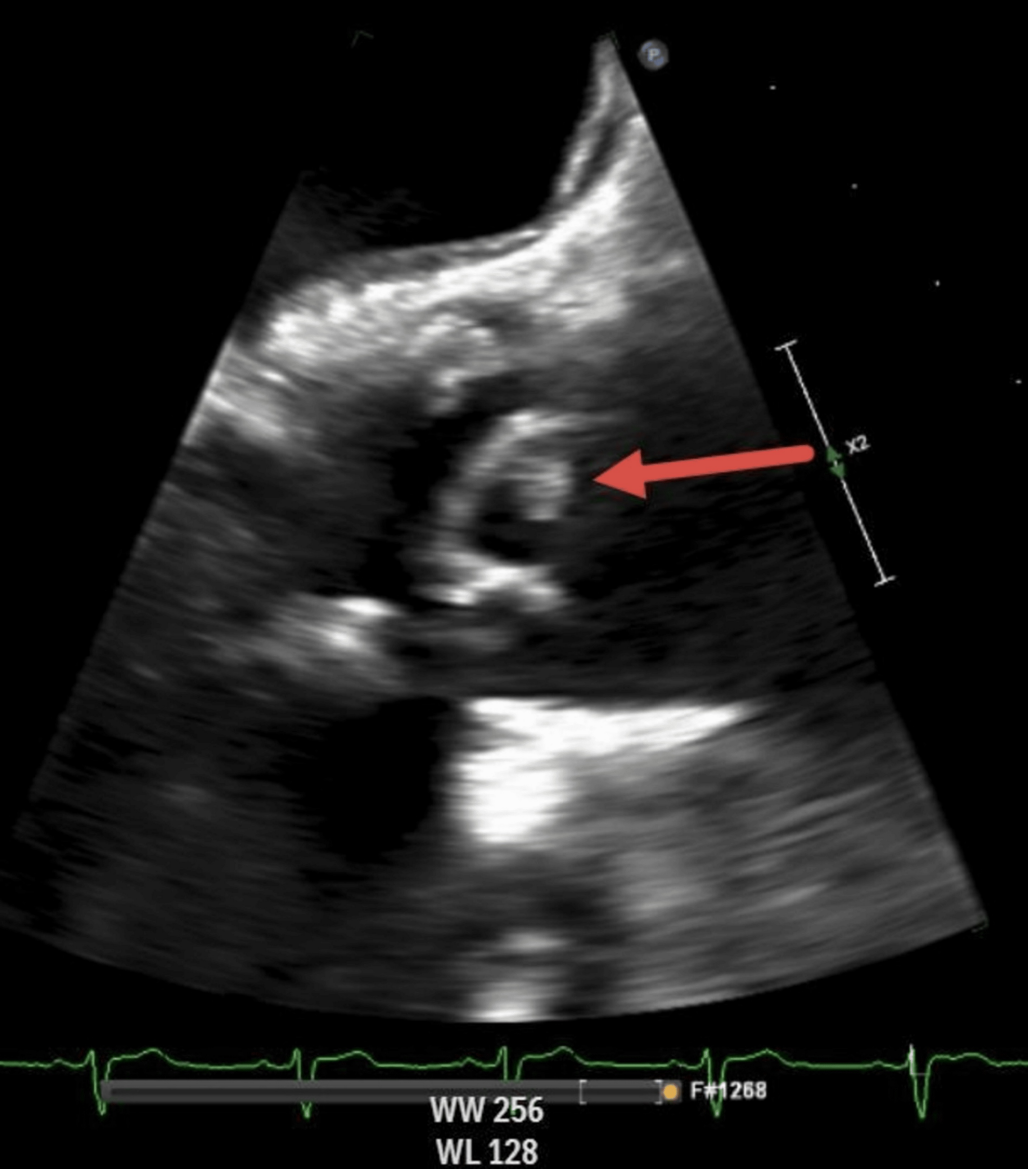
Transesophageal echocardiogram Longitudinal view of approximately 0.5 cm vegetation (red arrow). Mobile echodensity on one of the leaflets of the bioprosthetic aortic valve is concerning for endocarditis.

Repeat blood cultures again grew *R. aeria* following 48 hours of standard incubation. Due to our patient’s persistent, worsening symptoms and the continued isolation of *R. aeria* on blood cultures, there was high suspicion for infective endocarditis. An urgent transesophageal echocardiogram (TEE) revealed a mobile echodensity on one of the leaflets of the bioprosthetic aortic valve consistent with a valvular vegetation (Figure 1).

Due to the recent history of dental work, computed maxillofacial tomography was done, which failed to show an infectious process. A hepatitis panel was negative for the presence of hepatitis B and C infections, and a right upper quadrant ultrasound was unremarkable.

Given the fact that 4/4 subsequent blood cultures, drawn several days apart, continued to grow *R. aeria*, there was a true concern for invasive bacteremia with involvement of the prosthetic valve. The patient was started on broad-spectrum antibiotics with vancomycin 1,000 mg IV q12 hours, which was subsequently tailored to IV penicillin 4 million units q6 hours based on sensitivity results (Table 1).

**Table 1:** Antibiotic sensitivity chart of R. aeria in the index patient S: susceptible, NS: non-susceptible, I: intermediate, R: resistant

Specimen source	Blood	Arterial blood
Organism	Rothia aeria	Rothia aeria
Ciprofloxacin	<=1 S	<=1 S
Clindamycin	1 I	2 I
Daptomycin	2 NS	2 NS
Erythromycin	<= 0.500 S	<= 0.500 S
Gentamicin	<=4 S	<=4 S
Linezolid	<=1 S	<=1 S
Penicillin	<=0.120 S	<=0.120 S
Trimethoprim/sulfamethoxazole	<=2 S	<=2 S
Tetracycline	<=2 S	<=2 S
Vancomycin	1 S	1 S

As represented in Table 1, the patient’s* R. aeria *growth was inhibited by low amounts of penicillin concentration, making it the best antibiotic choice for this sample. There are limitations to using one static outcome to base the decision of the antibiotic regimen; therefore, continuous clinical monitoring was vital. Repeat surveillance blood cultures drawn after the initiation of antibiotics were all negative during hospitalization. Cardiothoracic surgery evaluated the patient and recommended conservative management for* Rothia* prosthetic valve endocarditis. The patient improved clinically and was discharged with a recommendation for six weeks of IV penicillin 24 million units q24 hours with two weeks of IV ceftriaxone 2 g IV q24 hours, which was included out of concern for possible biofilm formation on the prosthetic valve. Rifampin was initially considered but was deferred due to elevated transaminases throughout admission.

Repeat cultures and TEE were performed at the completion of antibiotic therapy, which demonstrated bloodstream clearance and interval reduction of valvular vegetation. The patient continues to have close follow-up outpatient visits to assess the long-term risk of* R. aeria* endocarditis.

## Discussion

Based on the current literature, there are nine documented cases of *R. aeria* causing native valve endocarditis (NVE) [1-9]. These documented cases have a variety of clinical presentations. A few cases presented with cerebral involvement, brain hemorrhages, cerebral mycotic aneurysms, or multiple systemic embolisms. Although all presented uniquely, what they have in common is the utilization of MALDI-TOF MS in the identification of *Rothia *spp. Herein, we report the first case of *R. aeria* prosthetic valve endocarditis (PVE) in an immunocompetent host.

*Rothia *spp. are encapsulated, non-acid-fast, non-motile, non-sporogenic, aerobic or facultative anaerobic rods [7]. Their morphology varies from coccoid forms (typically observed in broth culture media) to filamentous forms (typically observed on agar plates) [11]. This genus grows well under aerobic conditions on Brain Heart Infusion (BHI) agar. Young colonies are often smooth but tend to become rough, dry, convex, and adherent to the culture medium when mature. The *Rothia *spp. are commensal organisms of the mouth and upper respiratory tract flora. There are a total of 15 subspecies of *Rothia*, three of which (*R. mucilaginosa*,* R. dentocariosa*,* *and *R. aeria*) are currently viewed as potential human pathogens [12,13]. Infections with these subspecies have been reported in immunocompromised hosts, particularly in patients with hematological malignancies. Of the *Rothia *spp.*, R. dentocariosa* is most likely to cause infective endocarditis [9].

In a systematic review conducted by Franconieri et al., endocarditis caused by *Rothia* spp. is rare, with more than 60% of patients being immunocompromised [11]. *Rothia aeria* is not uncommonly misidentified as a Gram-positive bacillus, such as *Actinomyces* or *Nocardia*; Gram-positive cocci, such as *Staphylococcus*, or *Micrococcus* spp. [11]. In addition, *R. aeria* can be mislabeled as *R. dentocariosa*, given that both organisms have a 16S rRNA sequence similarity of 99.8%. It is due to the advent of the MALDI-TOF MS Brucker that *R. aeria* can nowadays be easily distinguished from* R. dentocariosa *[11]. Therefore, *R. aeria *is now being identified in previously unseen clinical settings. The increase in utilization and training in MALDI-TOF MS allows for proper identification of underdiagnosed and possibly virulent organisms.

*Rothia aeria* is an opportunistic organism that has been noted to cause invasive disease in immunocompromised hosts [5]. Until recently, the literature showed that *R. aeria *was mainly limited to periodontal infections. However, there is now a growing body of evidence that *R. aeria* can be the primary driver in conditions such as bacteremia, osteomyelitis, pneumonia, skin and soft tissue infections, and native valve endocarditis [14-16].

Its ability of biofilm formation is behind its main virulence factor that facilitates infection of native cardiac valves, such as mitral and aortic valves [1-3,5-11,17,18]. Although we only found one documented instance of *R. dentocariosa* as the culprit for prosthetic valve endocarditis (PVE) affecting the pulmonic valve, there are otherwise no known reports of *R. aeria *causing PVE [17]. Therefore, deciding whether *R. aeria* on blood cultures represents a true infection or a contaminant can be difficult, which can, in many cases, lead to a delay in treatment.

Our patient had a recent vigorous, deep cleaning dental procedure, eight weeks before the onset of symptoms. This dental procedure was a risk factor for his bacteremia and resulting infective endocarditis, despite the use of prophylactic antibiotics. Generally, committees universally agree that antibiotic prophylaxis is permitted for high-risk individuals undergoing high-risk invasive dental procedures that include gingival manipulation or manipulation of the apical region of teeth, with risk of mucosal perforation. According to Dayer et al., the effectiveness of antibiotic prophylaxis to prevent infective endocarditis is primarily derived from longitudinal studies, and while these studies suggest that complete cessation of antibiotic prophylaxis may increase the risk of endocarditis, they are underpowered and have limitations [18]. Given that our patient received prophylaxis per American Heart Association (AHA) guidelines, due to his surgical history of having a prosthetic aortic valve and still developed endocarditis, we can suggest more focused and better powered studies are needed in providing clearer data in regard to which patient populations should be targeted in receiving antibiotics in addition to highlighting if the short duration of antibiotics is sufficient to prevent infection [18].

Franconieri et al. showed that 97% of isolates were sensitive to penicillin and 92% to rifampicin [11]. *Rothia *spp.are often resistant to daptomycin, as high minimum inhibitory concentration (MIC) levels have been reported for *R. aeria *[11,15]. In three of the cases reported in the literature, treatment was changed to monotherapy with an aminopenicillin or ceftriaxone after initial empiric coverage with a beta-lactam antibiotic [1,5,6]. In a case described by Greve et al., high-dose intravenous penicillin was used for three weeks, which was later changed to IV ceftriaxone for an additional three weeks in an outpatient setting [17]. Based on this recommendation, we proceeded to treat our patient with a course of IV penicillin 24 million units q24 hours for six weeks to serve as the backbone of therapy and with IV ceftriaxone 2 g daily for two weeks with the intent of preventing biofilm formation, which was illustrated in the study by Greve et al. [17]. While our team did consider PO rifampin as adjunctive therapy for an organism with biofilm capabilities, the patient’s liver enzymes were above normal range; therefore, this course of therapy was contraindicated, and we proceeded with the aforementioned regimen. Repeat cultures and TEE confirmed bacterial clearance and reduction in the size of vegetation, respectively.

Patients with *R. aeria* native valve endocarditis (NVE) can expect a good outcome following an appropriate regimen and duration of antibiotics. In a case series of Aoyagi et al., seven out of nine patients recovered uneventfully [9]. This likely reflects the high susceptibility profile of *R. aeria* to a variety of antibiotics.

## Conclusions

Our case represents the first case of PVE due to *R. aeria* in an immunocompetent host. It raises awareness of *R. aeria* as a potential bloodstream pathogen. In addition, our case adds to the literature that *Rothia *spp., as a whole, has the potential to cause both native and prosthetic valve endocarditis. Due to the small number of reported invasive *Rothia* infections of heart valves and given the lack of MIC cutoff values, antibiotic therapy is chosen based on in vitro susceptibility testing. However, the reported susceptibilities of *R. aeria* to beta-lactam antibiotics and the current Repeat Infective Endocarditis (RIE) guidelines would suggest that a high dose of penicillin derivative for six weeks may represent a new standard of treatment for *R. aeria* infective endocarditis. However, this conclusion is drawn from observation of a limited number of cases. This illustrates the necessity for further research to ascertain optimal treatment regimens, address the variability in *Rothia* infections, create recommendations for similar cases, and determine long-term outcomes of *Rothia*-associated endocarditis. Prompt diagnosis and aggressive treatment are imperative in order to ensure the best clinical outcomes in the affected patients.
